# Myxoid Liposarcoma of the Thigh in Early Puerperium—Rare Case Report and Narrative Review

**DOI:** 10.3390/diseases13070220

**Published:** 2025-07-14

**Authors:** Tomasz Machałowski, Piotr Gutowski, Edyta Zagrodnik, Aleksandra Godlewska, Katarzyna Śmieja, Oliwia Kawałek, Anna Grzymała-Figura, Sylwester Michał Ciećwież, Katarzyna Gross-Kępińska, Małgorzata Szczuko, Maciej Ziętek

**Affiliations:** 1 Department of Perinatology, Obstetrics and Gynecology, Pomeranian Medical University, 70-204 Szczecin, Poland; tomasz.machalowski@pum.edu.pl (T.M.); aleksandra.godlewska@pum.edu.pl (A.G.); katarzyna.smieja@pum.edu.pl (K.Ś.); oliwiakawalek@gmail.com (O.K.); anna.grzymala.figura@pum.edu.pl (A.G.-F.); sylwester.ciecwiez@pum.edu.pl (S.M.C.); 2 Department of Vascular Surgery and Angiology, Pomeranian Medical University, 70-204 Szczecin, Poland; piotr.gutowski@pum.edu.pl; 3 Clinical Department of Anesthesiology and Intensive Care of Adults and Children, Pomeranian Medical University, 70-204 Szczecin, Poland; edyta.zagrodnik@pum.edu.pl; 4 INVICTA Fertility and Reproductive Center, 80-850 Gdańsk, Poland; katarzyna.gross-kepinska@invicta.pl; 5 Department of Bromatology and Nutritional Diagnostics, Pomeranian Medical University in Szczecin, 71-460 Szczecin, Poland; malgorzata.szczuko@pum.edu.pl

**Keywords:** myxoid liposarcoma, pregnancy, puerperium, sarcoma

## Abstract

Background: Liposarcoma (LPS) is a rare malignant tumor, but it is also one of the most common adult soft-tissue sarcomas. Myxoid liposarcoma (MLPS) accounts for 30% of all LPS cases. Diagnosis during pregnancy and the puerperium is very rarely reported, and only a few cases have been reported in the thigh. Case presentation: We report the case of a 36-year-old female patient on the 11th day of the puerperium after a cesarean section. The patient presented to the gynecology ward owing to the sudden appearance of a tumor in the medial part of her right thigh. The lesion was non-painful, mobile, soft, approximately 20 cm in diameter, and protruded above the level of the rest of the thigh surface. A suspicion of hematoma was raised. The final diagnosis was high-grade MLPS. Conclusions: An MLPS diagnosis is uncommon in female patients and even rarer during pregnancy. This case represents a novel occurrence, as the first instance in which symptoms manifested during the puerperium. Proper treatment and early detection could improve disease outcomes.

## 1. Introduction

Sarcomas represent a minority of all cancers, having a prevalence of less than 1% [1]. Liposarcoma is a prevalent form of soft-tissue sarcoma that occurs most commonly in adults, characterized by a variety of histopathological subtypes [2]. Liposarcoma originates from cells that build adipose tissue [2,3]. The prognosis of liposarcoma depends on the tumor subtype, location, and stage at the time of diagnosis, and early diagnosis is very rare if the tumor remains unpalpable for a long period [3,4,5]. Although this tumor can grow anywhere on the body, the abdomen, thigh, and popliteal fossa are typical sites for its occurrence [6]. This disease is classified into five principal subtypes: well-differentiated LPS (WDLPS), dedifferentiated LPS (DDLPS), myxoid LPS (MLPS), pleomorphic LPS (PLPS), and myxoid pleomorphic LPS (MPLPS) [2,3,4,7,8]. This group of cancers is heterogeneous, and depending on the subtype, they differ in clinical presentation, disease course, prognosis, and the age at which they develop [2,8]. LPS forms in adipocytes, where, as a result of mutations, intense cell division occurs, forming a tumor mass. Despite many studies, it is still not known what induces intensive cell divisions in fat cells. A higher incidence of soft-tissue sarcomas is associated with rare genetic syndromes in immunocompromised patients, such as neurofibromatosis types 1 and 2, Li and Fraumeni syndrome, Gardner syndrome, and Werner syndrome, as well as exposure to ionizing radiation and certain chemical agents, such as herbicides, pesticides, polyvinyl chloride, and dioxins [8,9,10,11].

MLPS accounts for 30% of all LPS cases [3,12,13]. It usually affects younger patients (between 30 and 50 years old) more than other subtypes. Some diagnoses are made in childhood or during adolescence, and it is more common in men [14]. Indeed, this diagnosis is rare in female patients and extremely rare during pregnancy (less than 20 cases in the English literature). Some of the diagnoses made during pregnancy refer to the thigh [13,15,16] and the retroperitoneum [5,16,17,18] (Table 1). The retroperitoneal area is the most common location for patients during pregnancy [1]. The main treatment option for localized liposarcoma is currently radical surgical resection, very often combined with radiotherapy. Surgery centralization is the best strategy for reducing the risk of death and relapse. The first-line treatment for unresectable sarcoma and disease with metastasis is chemotherapy based on anthracycline, but unfortunately, the chemosensitivity of liposarcomas remains very low [3,11,12].

In this study, we report the first case in the English literature of myxoid liposarcoma in the thigh during the puerperium. Previously, such cases have only been described during pregnancy [13,16]. The main clinical manifestation was the sudden (i.e., overnight, on day 12 of the puerperium) appearance of a tumor in the proximal part of the thigh, in the long adductor muscle.

## 2. Case Report

A 36-year-old pregnant woman at 38 + 1 of her third pregnancy presented at the maternity ward for delivery. The course of the pregnancy to date had been uncomplicated. The patient had 10 gynecological outpatient clinic visits and three standard fetal ultrasound examinations. No abnormalities were found during subsequent obstetric visits, and laboratory tests showed no deviation from normal values. The patient had a history of two pregnancies. The first pregnancy ended at term with a cesarean section due to the risk of intrauterine fetal distress during birth. Two years later, the patient presented with a second pregnancy, which also ended in a cesarean section due to a thin lower uterine segment after the prior cesarean section and suspected lower uterine segment distention at the scar site. Both cesarean sections were uncomplicated, and the patient left the hospital on postoperative day 3 in both cases (Table 1). Upon admission to the hospital, the patient reported no complaints. CTG records confirmed fetal well-being with a baseline fetal heart rate of 140/min. Fetal ultrasound showed a eutrophic fetus with an expected weight of 3300 g, normal amniotic fluid volume, and normal Doppler velocities in the umbilical artery and middle cerebral artery. The lower uterine segment was 1.8 mm thick, with intact continuity. Considering the two previous cesarean sections, it was decided to effect delivery via elective cesarean section. The operation was uncomplicated, and a son weighing between 3380 g and 2980 g was born. The patient left the hospital on the third postoperative day, and the initial puerperium was uncomplicated.

**Figure 1:**
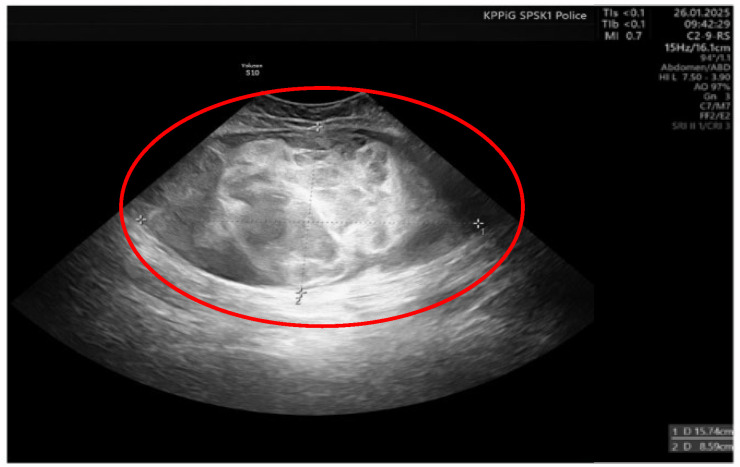
Ultrasound image of right thigh tumor (GE Voluson S10 Expert; BT16, 2019; 3/4D convex probe RAB6-RS) Red circle- tumor.

On the 11th day of the puerperium, the patient presented at the gynecology ward again during overnight hours due to the sudden appearance of a tumor in the medial part of the right thigh. The lesion was non-painful, mobile, soft, approximately 20 cm in diameter, and protruded above the level of the rest of the thigh surface. It was not accompanied by visible skin lesions. Owing to the sudden onset and clinical presentation of the lesion, a preliminary diagnosis of soft-tissue hematoma during the rupture of a venous vessel was made. An ultrasound examination (GE Voluson S10 Expert; BT16, 2019; convex 3/4D RAB6-RS transducer; General Electric Company, Schenectady, USA) showed an oval, focal, and confined space of variable echogenicity, measuring 15.74 × 8.59 cm, with a superficial localization involving the soft tissues of the right thigh and the lower pole reaching the quadriceps muscle (Figure 1).

**Figure 2:**
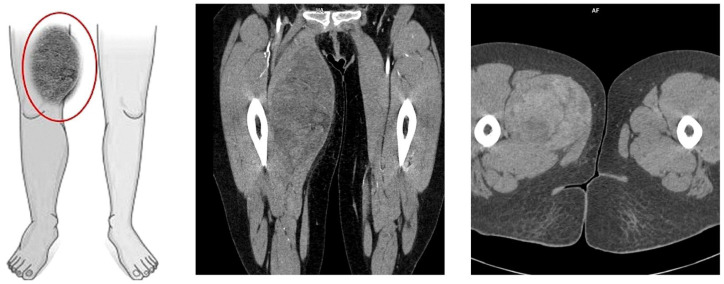
Picture of the tumor’s location and axial/coronal computed tomography imaging of a massive right thigh tumor—red circle (SOMATOM Edge Plus 128-layer CT scanner).

On the following day, computed tomography of the lower limb was performed. In almost the entire adductor muscle of the right thigh (the proximal part of the thigh, in the long adductor muscle), a large, well-demarcated, heterogeneous area measuring 96 × 86 × 175 mm (RL × AP × CC) was visible, showing a density of −5–22 H.U. in the native phase, with areas undergoing heterogeneous post-contrast enhancement up to 70 H.U. in the venous phase and 100 H.U. in the delayed phase. The image suggested a hematoma, and contrast enhancement in subsequent examination phases suggested active bleeding without a bleeding site detectable in a CT scan (Figure 2). For this reason, the patient was transferred to the Clinic of General and Vascular Surgery for surgical treatment and to manage the suspected bleeding.

**Figure 3:**
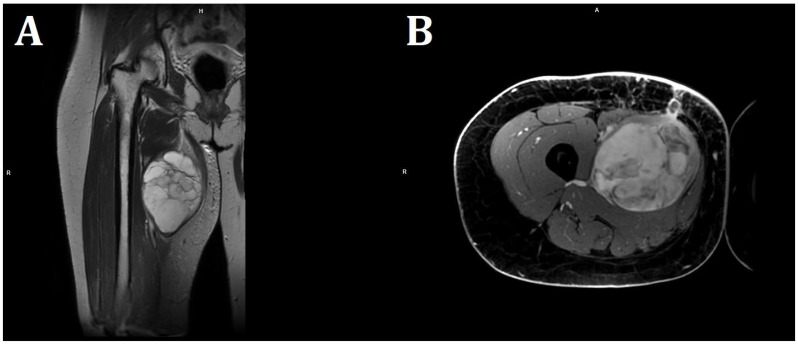
MRI imaging of a massive right thigh tumor. (**A**) shows a coronal T2-weighted image, and (**B**) shows an axial contrast-enhanced T1-weighted image. The signal intensity of the tumor is very bright on the T2-weighted image (**A**), suggesting that the tumor contains myxoid parts. The contrast-enhanced T1-weighted image shows a strong contrast enhancement of the tumor (**B**) (Philips MR 7700, 3.0 T; Philips, Cambridge, USA).

Empirical antibiotic therapy and compression therapy with elevation of the lower limb were introduced into the treatment. On the following day, the patient was qualified for open surgery performed by an experienced vascular surgeon. After an incision of the skin at the tumor level, at a length of 20 cm, an abundant mucous mass of about 600 g was detected infiltrating the quadriceps muscle of the thigh. No hematoma was found. The abnormal mucinous tissue was removed, and the thigh wound was sutured, leaving a drain. The procedure was uncomplicated, but the lesion was not removed in its entirety due to deep infiltration of the quadriceps muscle. In magnetic resonance imaging (MRI) of both thighs after surgical treatment, in the proximal part of the thigh, in the long adductor muscle, a nodular lesion measuring 138 × 89 × 71 mm having numerous internal septa was visible. The lesion was heterogeneous, with an increased signal in T2-weighted and PD-weighted images (Figure 3).

The histopathology in Szczecin (the local clinical hospital) demonstrated a low-grade MLPS with positive surgical margins. IHC results showed the following: vimentin (+), S-100 (+), CD34 (−), CDK4 (−), MDM2 (−), P16 (+), and p53 (−). An MDM2 molecular FISH test was negative. A fluorescence in situ hybridization study indicated no amplification of the MDM2 gene in the tumor cells, and the MDM2 signal index/CEN12 was 1.1. The number of MDM2 signals/cell was 2.2. The cut-off point for a positive result was defined as an MDM2 index/CEN12 above 2. Given this diagnosis, the patient was qualified for further treatment in the Department of Soft Tissue/Bone Sarcoma and Melanoma Maria Skłodowska-Curie Memorial Cancer Center in Warsaw, the national reference center for the treatment of sarcomas, where almost all cases of sarcoma in Poland are treated.

**Figure 4:**
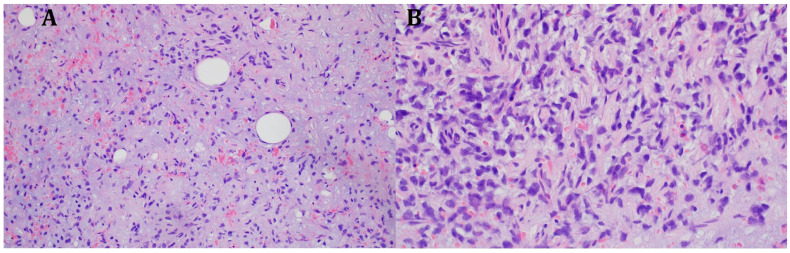
Microscopic image from the Department of Soft Tissue/Bone Sarcoma and Melanoma Maria Skłodowska-Curie Memorial Cancer Center in Warsaw. High-grade MLPS FNCLCC (*Federation Nationale de Centros de Lutte Contre le Cancer*) Grade 2 (G2). (**A**) H&E (hematoxylin and eosin steins) 20× (**B**) H&E 40×.

The histopathological blocks from the operation were then re-analyzed in Warsaw. A FISH DDIT3 test and an additional IHC test were ordered. In the assessment of rearrangements within the DDIT3 gene, 95% of the examined cells were confirmed to be neoplastic. Tumors having round cell components higher than 5% of all tumor volumes were considered high grade (9% in this case). The below image (Figure 4) indicates rearrangements within the DDIT3 gene (12q13). In addition, the IHC confirmed SOX 11 (+). The FISH test results confirmed the diagnosis of myxoid liposarcoma. Due to the focally increased cellularity of the lesion, the diagnosis was changed to high-grade MLPS FNCLCC (*Federation Nationale de Centros de Lutte Contre le Cancer*) Grade 2 (G2) (Figure 4). In a chest CT scan, MRI of both lower limbs, abdominal—pelvic CT, and MRI of the whole body, there were no signs of metastases (M0 N0). The treatment plan changed during the preparation of this article. The first plan was to prepare the patient for a specific expert orthopedic oncology operation; however, after seeing the NGS and FISH results and changing the diagnosis to high-grade myxoid liposarcoma, an oncological committee review (tumor board review) was performed. This tumor board always includes a surgeon specializing in the relevant area, a clinical oncologist, a geneticist, a radiation therapist, and a chemotherapist. The decision was to perform neoadjuvant radiotherapy and chemotherapy before the operation (Figure 3). The patient gave written consent to publish the description of her case.

## 3. Discussion

This case is the first to present with its first symptoms in the postpartum period and was diagnosed only recently. This disease originates in the proximal limbs, with about 75% of cases in the thigh and sometimes the retroperitoneum (Table 2) manifesting as a large, painless mass in deep soft tissues [1,3,6,12,13]. MLPS is aggressive, with local recurrence reported in up to 75% of cases and mortality at 15–30% [2,7,12,16]. MLPS can disseminate into distant sites and metastasize in up to 40% of all cases. The most common sites of metastases are serosal membranes, like the peritoneum, pericardium, pleura, abdominal cavity, and bones [3]. Interestingly, in our case, the disease was completely asymptomatic throughout the pregnancy, and in the first days of the postnatal period, there were no bowel problems, pain, movement disorders, or lethargy as in other cases described [10,16]. Thus, we suspected an asymptomatic hematoma caused by a spontaneous rupture of a venous vessel in the thigh, possibly associated with a history of cesarean sections. During examination, CT, angio CT, and MRI are reliable and important tools for diagnosing MLPS. Moreover, these methods are very important for obtaining prebiopsy information [13].

In the CT scan large, well-demarcated tumor density was −5–22 H.U. in the native phase, with areas undergoing heterogeneous post-contrast enhancement up to 70 H.U. in the venous phase and 100 H.U. in the delayed phase. Retrospectively, it is possible to consider a tumor with a lipid component when H.U. is less than 0 [14]. On the other hand, Hassan et al. suggested that most hematomas (64%) had mixed density with hyperdense components [19], and fat components typically have between −65 and −120 H.U. [20] Perhaps this was the reason why two specialists in radiology and diagnostic imaging suggested, based on this description and the images, that it could be a hematoma.

The gold standard for diagnosis remains histopathological material from needle biopsies or surgery, and a very important element in determining the type of LPS is molecular testing [2]. More than 90% of MPLS cases have a pathognomonic translocation at t (12;16) (q13;p11), and about 5–10% of cases will have a translocation at t (12;22) (q13;q12), expressing FUS-DDIT3 fusion proteins; this was confirmed in our patient through her immunohistochemistry. The vast majority of myxoid liposarcomas exhibit no *DDIT3* expression [4,6,14,18,21]. The genes localized in damaged regions contributing to MPLS pathogenesis include *TP53, RB1* (13q14), and *ATRX* (Xq21 [22,23,24]). These modifications induce cell cycle dysregulation, chromatin remodeling abnormalities, and tumor advancement in MPLS. Recurrent TP53 mutation was identified in 100% of patients (8 out of 8) in recurrent cases of MPLPS [24]. In an article by Creytens et al., chromosomal losses on chromosome 13, namely at 13q14 (including the *RB1*), were identified in 4 of the 8 instances examined [23]. There was no *DDIT3* gene rearrangement or *MDM2* gene amplification, and this can be utilized to differentiate this variant of liposarcoma from others [22,23].

A promising noninvasive method for prognosis, therapy response, and disease monitoring is circulating tumor DNA (ctDNA [25,26]). The identification of mutations and structural variations unique to sarcoma has been made possible by advancements in detection techniques, such as PCR, next-generation sequencing (NGS), and low-pass whole-genome sequencing [25,26,27]. ctDNA is a new potential marker of disease progression and remission in sarcoma. Targeted NGS is a very useful tool in potentially moving patients into mutation-specific clinical trials. It is also necessary to determine if mutations are clinically meaningful targets for drugs in sarcoma [27]. In the diagnostic setting, ctDNA facilitates the identification of characteristic structural variants, translocations and epigenetic alterations, thereby offering molecular insights that augment conventional tissue biopsies and overcome the obstacles presented by tumor heterogeneity and restricted biopsy accessibility [25,26,27]. Across several sarcoma subtypes, ctDNA levels exhibit a substantial correlation with tumor burden, treatment response, and recurrence risk when it comes to disease monitoring [25]. CtDNA potentially provides more sensitivity than imaging in detecting relapses early and detecting minimum residual illness, which could enable preventative treatment therapies, but there is still a lack of extensive clinical validation [25,26,27]. There are still a number of issues that hinder its broad clinical use. In general, sarcomas shed low amounts of ctDNA detection, and its usefulness as a biomarker is limited. Tackling this challenge highlights the urgent need for more precise, reliable, and affordable products and services [25,27] Future research should focus on prospective validation in clinical trials, the standardization of assay methodologies, and integration into risk-adapted treatment algorithms The standardization of assay procedures, incorporation into risk-adapted therapy algorithms, and prospective validation in clinical trials should be the main areas of future study [25,26].

The main treatment option is surgical, and the complete resection of the mass must be the goal. After surgical treatment, patients require outpatient follow-up given the high risk of distant and local recurrence [1,10,13,28]. A rare symptom in an obstetric woman is a thigh lesion, and our patient’s situation shows that even if there is a logical explanation for a possible hematoma, the first option that should be considered is surgery with the aim of complete removal of the lesion. We would like to avoid situations of R2 (macroscopic residual tumor). Radiotherapy is an effective treatment for patients with unclear surgical margins or locally advanced sarcoma, as in the case of our patient. MLPS is particularly radiosensitive compared with other liposarcomas [1,3,4,5,13]. To reduce the recurrence rate, adjuvant chemotherapy is currently proposed (local and distant) in the high-risk patient group [1,3,4,12,13].

The step after NGS sequencing and ctDNA diagnostics was three cycles of neoadjuvant chemotherapy and one cycle of neoadjuvant radiotherapy. The aim was to prepare the patient for radical surgery and to avoid repeating the surgical problem due to R2. The plan after this treatment and re-evaluation of ctDNA and repeat MRI was surgical treatment by a qualified team of orthopedic oncologists, along with oncological surgeons at the Department of Soft Tissue/Bone Sarcoma and Melanoma Maria Skłodowska-Curie Memorial Cancer Center in Warsaw (Figure 4). The plan is to perform a limb-sparing surgery, for which the application of neoadjuvant systemic therapy will be helpful. Of course, in case of progression of the disease, there could be amputation of the limb.

The neoadjuvant chemotherapy protocol was three cycles in the AI regimen (ifosfamide and doxorubicin), and infusion on days 1–3 or continuous drug infusion for 72 h with a total dose of 5 g/m^2^ of ifosfamide and 60 mg/m^2^ of doxorubicin per cycle. A multicenter phase II clinical trial SARC006 (NCT00304083) was conducted comparing the efficacy of neoadjuvant chemotherapy using doxorubicin, etoposide, and ifosfamide. Patients undergoing neoadjuvant chemotherapy could undergo radical sparing treatment (radiotherapy or surgery) [29]. Moreover, the AI regimen was associated with the highest rate of objective responses. Additionally, it was very important for our patient, because the plan is to perform limb-sparing surgery. Patients who received a regimen with doxorubicin and ifosfamide achieved longer PFS (progression-free survival) compared to those treated with anthracycline monotherapy, while those treated with ifosfamide monotherapy had the shortest PFS [29]. Patients who received a regimen with doxorubicin and ifosfamide achieved a longer PFS compared to those treated with anthracycline monotherapy, while those treated with ifosfamide monotherapy achieved the shortest PFS [29].

**Figure 5:**
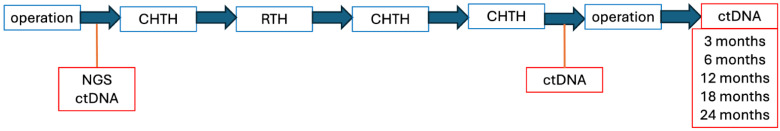
Individual therapeutic intervention scheme.

Next, to assess the potential recurrence of the disease, an MRI scan of the abdominal cavity or lower limb and a CT scan of the chest will be performed every 6 months. Additionally, the ctDNA will be evaluated every 3, 6, 12, 18, and 24 months (Figure 5).

New therapies that target specific epigenetic alterations in malignant tumors can be developed by analyzing immunohistochemistry and molecular genetics. Precise molecular studies and immunohistochemistry may have a significant impact on the effectiveness of the prognosis and further treatment of patients [10]. The most positive outcomes have been shown for MDM2 and CDK4/CDK6 inhibitors and also the multi-kinase inhibitors sinitinib and anlotinib [2,4,6]. In our case, the MDM2 and CDK4/6 IHC were negative. This means that these inhibitors will not be the targeted treatment. Estrogen and androgen receptors are highly expressed in liposarcoma, especially in well-differentiated cases. This information warrants further functional studies to identify receptor function in liposarcoma and its potential role in anti-hormone therapy [30,31]. With good treatment, the 5-year overall survival rate for our patient is about 78%. The risk for recurrence in similar anatomical locations with R2 is 50–75%. According to a comprehensive review of cases similar to that of our patient, the locations of the first metastases are soft tissues in 32% of patients, intra-abdominal in 26%, pulmonary in 24%, and bone in 17% [9,14,32,33,34,35].

Patients having a low BMI (BMI 23.8 in the case of our patient) may have an increased risk of soft-tissue sarcomas. Factors that can reduce the risk of soft-tissue sarcomas include shorter stature at puberty; increased parity (>3); and shorter contraceptive use, especially at a young age (none were the case for our patient). Growth hormones may also play an important role in the development of LPS [1,36].

The prognosis of this diagnosis depends on several factors, such as the histologic type of the tumor and its location, size, and degree of malignancy. In general, liposarcomas have a better prognosis than other soft-tissue sarcomas in adults. Well-differentiated liposarcoma, the most common type, yields high survival rates. The prognosis is favorable, especially when the tumor is located in the extremities. However, myxoid liposarcoma tends to recur locally and metastasize to serous surfaces and bones, especially the spine. The prognosis is moderate, with a 5-year survival rate of about 70–90%. Pleomorphic liposarcoma, the rarest and most malignant type, is located mainly in the lower extremities and has the worst prognosis, with a 5-year survival rate of about 40%. Tumor location is also a factor influencing prognosis. Tumors located in the lower extremities are associated with a better prognosis than in other locations. However, tumors larger than 5 cm are associated with a worse prognosis. Another element of prognostic significance is the completeness of tumor removal, which is associated with better treatment outcomes [37]. Based on a study by Koseła-Paterczyk H and Wągrodzki M, the clinical characteristics of LPS are shown in Table 3 [14].

In conclusion, due to the extremely rare occurrence of postpartum liposarcoma in young women, its definitive diagnosis is usually delayed. In the differential diagnosis of soft-tissue tumors of the thigh, hematoma, lipoma, and abscess are most commonly considered. The diagnosis of a soft-tissue malignancy can be guided by the absence of clinical signs of pain, redness, and inflammatory infiltration with an obviously palpable soft tumor protruding above the skin surface. Therefore, it is essential to carry out imaging (MRI, ultrasound, and CT) and molecular studies on the tissue material taken during surgery to make a definitive diagnosis and determine further treatment.

## Figures and Tables

**Table 1:** The medical and clinical characteristics of the patient.

Initials, Age	K.S., 36 Years Old
Weeks of gestation, Pregnancy, Delivery	38 + 4 weeks of the gestation, III pregnancy, III delivery
Chronic diseases	none
Operations	2 times c-section Reasons c-section: (year 2020—40 weeks of gestation, the risk of intrauterine fetal distress during birth, 3050 g) (year 2022—39 weeks of gestation, thin lower uterine segment after the prior cesarean section and suspected lower uterine segment distention at the scar site, 3130 g)
Hospitalization and complications during pregnancy	none

**Table 2:** Clinicopathological characteristics associated with pregnancy liposarcoma [1,5,13,15,16,17,18].

Author	Age	Complain	Gestation at Diagnosis	Location	Surgery	Prognosis
Kasashima et al., 2015 [5]	34	Abdominal distension	After delivery	Retroperitoneum	9 months after delivery	Free of disease 3 years postop.
Kasashima et al., 2015 (case 2) [5]	41	Weight loss	34	Retroperitoneum	36 weeks of gestation	Died after 8 months
Huo et al., 2015 [17]	27	Abdominal pain	16 weeks	Retroperitoneum	20 weeks of gestation	Alive 6 months after operation
Nguyen et al., 2024 [16]	27	Abdominal pain	15 weeks	Retroperitoneum	15 weeks	Remission 12 months after operation
Matsuda et al., 2000 (case 1) [15]	28	Pain in left thigh	29 weeks	Left thigh	RTH before operation, surgery 5 weeks after delivery	Free of disease 5 years postop.
Matsuda et al., 200 (case 2) [15]	34	Large mass in right thigh, recurred 3 months after operation	20 weeks	Right thigh	Termination of pregnancy at 33 weeks of gestation (rapid growth of tumor), surgery after delivery	Free of disease 4 years postop.
Zhang et al., 2020 [13,18]	36	Mass in right hip growing for 4 months	25 weeks	Right hip	Termination of pregnancy at 37 weeks of gestation, OP+RTH (60G)	Free of disease 37 months after delivery
Ker et al., 2017 [1]	33	Painless, soft, elastic tumor in left thigh	18 weeks	Left thigh	18 weeks of gestation	Ongoing pregnancy

**Table 3:** Clinical characteristics of liposarcoma (LPS) [14].

Subtype	Risk of Local Recurrence	Risk of Metastatic Disease	Sensitivity to Chemotherapy	Sensitivity to Radiotherapy
Well-differentiated	Low	Low	Low	Moderate
Dedifferentiaed	High	Low	Low	Moderate
Myxoid Low grade **High grade**	Low **Intermediate**	Low High	High **High**	High **High**
Pleomorphic	Intermediate	High	High	Intermediate

## Data Availability

Our study data contains personal information of patient and, therefore, is not available for sharing due to the ‘Personal Data Protection Law’ and ethical reasons.

## References

[1] Ker C.R., Chen C.C., Huang S.H., Kao C.N., Shen C.J. (2017). Myxoid Liposarcoma at Thigh during Pregnancy: A Case Report and Literature Review. SM J Sarcoma Res..

[2] Lesovaya E.A., Fetisov T.I., Bokhyan B.Y., Maksimova V.P., Kulikov E.P., Belitsky G.A., Kirsanov K.I., Yakubovskaya M.G. (2024). Genetic, Epigenetic and Transcriptome Alterations in Liposarcoma for Target Therapy Selection. Cancers.

[3] Resag A., Toffanin G., Benešová I., Müller L., Potkrajcic V., Ozaniak A., Lischke R., Bartunkova J., Rosato A., Jöhrens K. (2022). The Immune Contexture of Liposarcoma and Its Clinical Implications. Cancers.

[4] Haddox C.L., Riedel R.F. (2021). Recent advances in the understanding and management of liposarcoma. Fac. Rev..

[5] Kasashima H., Yamasaki Y., Morimoto Y., Akamaru Y., Yasumasa K., Kasugai T., Yoshida Y. (2015). A case of retroperitoneal liposarcoma after delivery with expression of estrogen receptor: Report of a case. Int. J. Surg. Case Reports..

[6] Lee A., Thway K., Huang P.H., Jones R. (2018). Clinical and Molecular Spectrum of Liposarcoma. J. Clin. Oncology..

[7] Sbaraglia M., Bellan E., Dei Tos A.P. (2021). The 2020 WHO Classification of Soft Tissue Tumours: News and perspectives. Pathologica.

[8] Choi J.H., Ro J.Y. (2021). The 2020 WHO Classification of Tumors of Soft Tissue: Selected Changes and New Entities. Adv. Anat. Pathol..

[9] Genevois A.-L., Carton M., Jean-Denis M., Cyrta J., Corradini N., Berlanga P., Chemin-Airiau C., Honore C., El Zein S., Defachelles A.-S. (2023). Leiomyosarcoma and liposarcoma in young patients: The national netsarc+ network experience. EJC Paediatr. Oncology..

[10] Sosnowska-Sienkiewicz P., Mańkowski P., Stadnik H., Dłubak A., Czekała A., Karczewski M. (2023). A Rare Case of Dedifferentiated Liposarcoma with Osteosarcomatous Differentiation-Diagnostic and Therapeutic Challenges. Diseases.

[11] Fiedorowicz M., Bartnik E., Sobczuk P., Teterycz P., Czarnecka A. (2018). Molecular biology of sarcoma. Oncol. Clin. Practice.

[12] Suarez-Kelly L.P., Baldi G.G., Gronchi A. (2019). Pharmacotherapy for liposarcoma: Current state of the art and emerging systemic treatments. Expert Opin. Pharmacother..

[13] Zhang Y., Wang Y., Zheng C., Li X., Cui Y., Chen G., Wang Q., Liu F., Liu C., Xu T. (2020). A giant myxoid/round-cell liposarcoma of the hip in a pregnant woman: A case report and literature review. Int. J. Clin. Exp. Med..

[14] Koseła-Paterczyk H., Wągrodzki M. (2018). Liposarcoma-spectrum of disease. Oncol. Clin. Pract..

[15] Matsuda S., Tanaka K., Harimaya K., Matsumoto Y., Sato H., Iwamoto Y. (2000). Treatment of myxoid liposarcoma in pregnancy. Clin. Orthop. Relat. Res..

[16] Zarkavelis G., Petrakis D., Fotopoulos G., Mitrou S., Pavlidis N. (2016). Bone and soft tissue sarcomas during pregnancy: A narrative review of the literature. J. Adv. Res..

[17] Huo D., Liu L., Tang Y. (2015). Giant retroperitoneal liposarcoma during pregnancy: A case report. World J. Surg. Oncol..

[18] Nguyen M.P., Pham P., Vu A., Nguyen D., Tran H. (2024). Massive, Retroperitoneal, Well-Differentiated Liposarcoma in Second-Trimester Pregnancy: A Report of a Rare Case. Cureus.

[19] Hasan D., Nikoubashman O., Pjontek R., Stockero A., Hamou H.A., Wiesmann M. (2022). MRI appearance of chronic subdural hematoma. Front. Neurol..

[20] Liu D., Li C., Chen L. (2013). Management of giant intermuscular lipoma of hips: A case report and review of literature. Mol. Clin. Oncol..

[21] Scapa J.V., Cloutier J.M., Raghavan S.S., Peters-Schulze G., Varma S., Charville G.W. (2021). DDIT3 Immunohistochemistry Is a Useful Tool for the Diagnosis of Myxoid Liposarcoma. Am. J. Surg. Pathol..

[22] Shen Y., Zhao L., Li A., Peng Q., Liu Q., Wang L. (2024). Rare myxoid pleomorphic liposarcoma: A case report and literature review. J. Clin. Pathol..

[23] Creytens D., Folpe A.L., Koelsche C., Mentzel T., Ferdinande L., van Gorp J.M., Van der Linden M., Raman L., Menten B., Fritchie K. (2021). Myxoid pleomorphic liposarcoma-a clinicopathologic, immunohistochemical, molecular genetic and epigenetic study of 12 cases, suggesting a possible relationship with conventional pleomorphic liposarcoma. Mod. Pathol. Off. J. United States Can. Acad. Pathol. Inc..

[24] Dermawan J., Hwang S., Wexler L., Tap W., Singer S., Vanderbilt C., Antonescu C.R. (2022). Myxoid pleomorphic liposarcoma is distinguished from other liposarcomas by widespread loss of heterozygosity and significantly worse overall survival: A genomic and clinicopathologic study. Mod. Pathol. Off. J. United States Can. Acad. Pathol..

[25] Aiyer S., Kim T.H., Collier K., Pollock R., Verschraegen C., Stover D.G., Tinoco G. (2025). Unlocking the Potential of ctDNA in Sarcomas: A Review of Recent Advances. Cancers.

[26] Darville-O’Quinn P., Gokgoz N., Tsoi K.M., Andrulis I.L., Wunder J.S. (2024). Investigating the Use of Circulating Tumor DNA for Sarcoma Management. J. Clin. Med..

[27] Cote G.M., He J., Choy E. (2018). Next-Generation Sequencing for Patients with Sarcoma: A Single Center Experience. Oncologist.

[28] Tirotta F., Sayyed R., Jones R., Hayes A. (2022). Risk factors for the development of local recurrence in extremity soft-tissue sarcoma. Expert Rev. Anticancer. Ther..

[29] Czarnecka A.M., Sobczuk P., Zdzienicki M., Spałek M., Rutkowski P. (2018). Malignant peripheral nerve sheath tumour (MPNST). Oncology in Clinical Practice.

[30] Ingram D.R., Dillon L.M., Lev D.C., Lazar A., Demicco E.G., Eisenberg B.L., Miller T.W. (2014). Estrogen receptor alpha and androgen receptor are commonly expressed in well-differentiated liposarcoma. BMC Clin. Pathol..

[31] Costea R., Vasiliu E., Zarnescu N., Hasouna M., Neagu S. (2011). Large thigh liposarcoma--diagnostic and therapeutic features. J. Med. Life.

[32] Zhao J., Du W., Tao X., Li A., Li Y., Zhang S. (2025). Survival and prognostic factors among different types of liposarcomas based on SEER database. Sci. Rep..

[33] Jo S.J., Kim K.S., Lee K.W., Park J.B., Choi Y.L., Yu J.I., Lee S.J., Choi D.I., Sung J.K. (2018). Distribution and survival of primary sarcoma in Korea: A single center analysis of 2017 cases. Korean J. Clin. Oncol..

[34] Qu G., Zhang C., Tian Z., Yao W. (2024). Diagnosis and Treatment of Myxoid Liposarcoma. Curr. Treat. Options Oncology..

[35] Homsy P., Böhling T., Seitsonen A., Sampo M., Tukiainen E., Blomqvist C. (2023). Patterns of Metastatic Recurrence of Genetically Confirmed Myxoid Liposarcoma. Ann. Surg. Oncol..

[36] Wagner P., Alvegård T., Ranstam J., Rydholm A., Vult von Steyern F., Olsson H. (2014). Oral contraceptive use, parity, and constitutional characteristics in soft tissue sarcoma: A Swedish population-based case-control study 1988–2009. Cancer Causes Control.

[37] Liang S.X., Howitt B., Blitz M.J., Bhuiya T., Thamasebi F., Sternchos J., Shih K. (2015). Primary myxoid liposarcoma of the ovary in a postpartum female: A case report and review of literature. Int. J. Gynecol. Pathol. Off. J. Int. Soc. Gynecol. Pathol..

